# Supplementary material for: The cysteine-rich virulence factor NipA of Arthrobotrys flagrans interferes with cuticle integrity of Caenorhabditis elegans
Source: Nat Commun. 2024 Jul 10;15:5795. doi: 10.1038/s41467-024-50096-4 (PMC11237121; doi:10.1038/s41467-024-50096-4)
Supplement: Supplementary file 1 — Supplementary Information [file 41467_2024_50096_MOESM1_ESM.pdf]

Supplementary information

The cysteine-rich virulence factor NipA of *Arthrobotrys flagrans* interferes with cuticle integrity of *Caenorhabditis elegans*

Jennifer Emser[1], Nicole Wernet[1], Birgit Hetzer[2], Elke Wohlmann[1] and Reinhard Fischer[1]*

running head: *C. elegans* infection with *A. flagrans*

Address: [1]Karlsruhe Institute of Technology (KIT) - South Campus
Institute for Applied Biosciences
Dept. of Microbiology
Fritz-Haber-Weg 4
D-76131 Karlsruhe, Germany
Phone: +49-721-6084-4630
Fax: +49-721-6084-4509
E-mail: reinhard.fischer@KIT.edu
Homepage: www.iab.kit.edu

[2]Max-Rubner-Institut (MRI) - Federal Research Institute of Nutrition and Food
Haid-und-Neu-Strasse 9
76131 Karlsruhe

* corresponding author

Suppl. Information Table 1: *A. flagrans* and *C. elegans* strains used in this study.

Strain	Genotype	Reference
A. flagrans		
sJM01	Δ*nipA*, pJM06	This work
sJM02	*gpdA(p)::nipA::lccC*$^{\Delta AS1\text{-}18}$ pJM07	This work
sJM42	*gpdA(p)::nipA*$^{\Delta AS1\text{-}16}$*lcc*$^{\Delta AS1\text{-}18}$ pJM68	This work
sVW10	*h2b(p)::h2b::GFP::h2b(t)* pJW04	[15]
sJM15	sVW10 x *nipA(p)::h2b::mCherry::tubA(t)* pJM10	This work
sJM05	*nipA(p)::nipA::gfp pJM01*	This work
sVWZ	*tubA(p)::lifeact::gfp::gluC(t)*	[15]
sNH30	*cyrA(p)::cyrA::GFP::gluC(t)*	[20]
sJM08	sVW07 x *nipA(p)::nipA::mCherry* pJM16	This work
sJM09	sNHH30 x *nipA(p)::nipA::mCherry* pJM16	This work
sJM43	WT x *cyrA(p)::cyrA::mCherry* pNH94	This work
sJM44	Δ*nipA* x *cyrA(p)::cyrA::mCherry* pNH94	This work
sJM50	sJM44 x *nipA(p)::nipA::GFP* pJM102	This work
sJM51	sJM44 x *nipA(p)::nipA*$^{Cys23/Ala}$*::GFP* pJM100	This work
sJM52	sJM44 x *nipA(p)::nipA* pJM103	This work
C. elegans		
N2	Wild type	University of Freiburg
GS2478	arIs37 I; dpy-20(e1282) IV; cup-8(ar466) V	*C. elegans* center, University of Minnesota
GOU2043	*arIs37 I; dpy-20(e1282) IV; cup-8(ar466)*	*C. elegans* center, University of Minnesota
KIT39	N2 x *eft-3(p)::mScarlet* pJM86	This work
KIT40	N2 x *eft-3(p)::nipA::mScarlet* pJM85	This work
KIT41	GS2478 x *eft-3(p)::mScarlet* pJM86	This work
KIT42	GS2478 x *eft-3(p)::nipA::mScarlet* pJM85	This work
KIT44	N2 x *dpy-7(p)::nipA::mScarlet* pJM81	This work

KIT45	N2 x *col-19(p)::nipA::mScarlet* pJM46	This work
KIT46	N2 x *col-19(p)::mScarlet* pJM72	This work
KIT48	N2 x *col-19(p)::nipA*$^{\Delta SP}$*::mScarlet* pJM36	This work
KIT49	N2 x *col-19(p)::nipA* pJM32	This work
KIT50	GS2478 x *col-19(p)::mScarlet* pJM72	This work
KIT51	GS2478 x *col-19(p)::nipA::mScarlet* pJM46	This work
KIT52	N2 x *dpy-7(p)::mScarlet* pJM91	This work
KIT53	N2 x *dpy-7(p)::mScarlet; col-19(p)::nipA* pJM91; pJM32	This work
KIT54	GOU2043 x *col-19(p)::nipA::mScarlet* pJM46	This work
KIT55	GOU2043 x *col-19(p)::nipA* pJM32	This work
KIT57	N2 x *col-19(p)::nipA*$^{Cys/Ala}$*::mScarlet* pJM92	This work
KIT58	N2 *eft-3(p)::nipA::mScarlet; unc-122(p)::GFP* pJM85; pNH79	This work

Suppl. Information Table 2: Plasmids used in this study.

Name	Description	Reference
pNH10	*gpdA(p)::cyrA::lccC*$^{\Delta AS1\text{-}18}$ *trpC(p)::hph::trpC(t) (hph)*	[20]
pNH79	*unc-122::GFP*	N. Wernet
pNH94	*cyrA(p)::cyrA::mCherry::gluC(t); tub(p)::G418*	N. Wernet
pJM06	*ΔnipA x pJEt1.2, hph*	This work
pJM07	*gpdA(p)::nipA::lccC*$^{\Delta AS1\text{-}18}$*; hph*	This work
pJM68	*gpdA(p)::nipA*$^{\Delta AS1\text{-}16}$*lcc*$^{\Delta AS1\text{-}18}$*; hph*	This work
pJM10	*nipA(p)::h2b::mCherry::tubA(t); hph*	This work
pVW23	*h2b(p)::h2b::mCherry::h2b(t); hph*	[20]
pJM01	*nipA(p)::nipA::gfp; hph*	This work
pNH21	*oliC(p)::cyrA::gfp::gluC(t); hph*	[20]
pJM08	*oliC(p)::nipA::gfp::gluC(t); hph*	This work
pJM16	*nipA(p)::nipA::mCherry::gluC(t); gpdA(p)::g418::trpC(t) (g418)*	This work
pNH53	*oliC(p)::broA::GFP; g418*	N. Wernet
pNH94	*cyrA(p)::cyA::mCherry::gluC(t), g418*	N. Wernet
pJM32	*col-19(P)::nipA*	This work
pJM36	*col-19(P)::nipA::mScarlet::unc-54 3'UTR*	This work
pJM46	*col-19(P)::nipA::mScarlet::unc-54 3'UTR*	This work
pJM72	*col-19(P)::mScarlet::unc-54 3'UTR*	This work
pJM81	*dpy-7(p)::nipA::mScarlet::unc-54 3'UTR*	This work
pJM85	*eft-3(p)::nipA::mScarlet::unc-54 3'UTR*	This work
pJM86	*eft-3(p)::mScarlet::unc-54 3'UTR*	This work
pJM91	*dpy-7(p)::mScarlet::unc-54 3'UTR*	This work
pJM92	*col-19(p)::nipA*$^{Cys23/Ala}$*::mScarlet::unc-54 3'UTR*	This work
pJM100	*nipA(p)::nipA*$^{Cys23/Ala}$*::GFP::gluC(t);* nat	This work
pJM102	*nipA(p)::nipA::GFP::gluC(t);* nat	This work
pJM103	*nipA(p)::nipA::gluC(t);* nat	This work

Suppl. Information Table 3: Oligonucleotides used in this study.

Name	Sequence 5'→ 3'	Description
5407_AscI_for	GGCGCGCCATGAAAGGCGCTATCCTCATTAT	C-terminal Laccase/GFP fusion
5407_noStop_AgeI_rev	TATACCGGTGGCTTCCTCGTCGGGGAT	C-terminal Laccase fusion
nipA_noSP_gpdA(p)_OL_fwd	agcagacatcacaGGCGCGCCATGCCGACCGTAAGTGCCCAAG	C-terminal Laccase fusion without SP
SB_5407_RB_fwd	GGATTTCCAGAAATCGCATGC	Southern-Blot analysis
SB_5407_RB_rev	GAGGCAATAGGTCCAATTTATAC	Southern-Blot analysis
nipA_noStopp_Lcc_OL_rev	TGCTCAAAGACCCAAGAACCGGTGGCTTCCTCGTCGGGGAT	C-terminal Laccase fusion without SP
5407_RT_for	ATG AAA GGC GCT ATC CTC ATT AT	qRT PCR
5407_RT_rev	CAA TTG GCG GAA TTC TTG CAT TT	qRT PCR
qpcr_hk_actin2_fwd	TCCAGACTGCCTCCCAGT	qRT PCR; Youssar
qpcr_hk_actin2_rev	AGGTCTTTTCTGACGTCGAC	qRT PCR; Youssar
p5407_H2B_OL_rev	CTTTTCGGCGGCGGCTTTTGGTGGCATTTTGTTGGGCTTTCTAACCAC	Promoter fusion
p5407_1.7_EfiOL_fwd	TGTAAAACGACGGCCAGTGAATTCGGCACTTTTGTGTAATTGCGCATTGG	Promoter fusion
Backbone_Efi_rev	GAATTCACTGGCCGTCGTTT	Backbone amplification
H2B_NLS_for	ATGCCACCAAAAGCCGCC	Backbone amplification for promoter fusion
5407_PacI_noStop_rev	gcgttaattaaGGCTTCCTCGTCGGGGAT	C-terminal GFP fusion
p5407_pJM08_OL_fwd	CCCTAAACTCCCCCCAGGCACTTTTGTGTAATTGCGC	C-terminal GFP fusion under native promoter
p5407_pJM08_OL_rev	GATAGCGCCTTTCATGGTTTGTTGGGCTTTCTAACCACAA	C-terminal GFP fusion under native promoter
pJM08_BB_5407_ fwd	CCATGAAAGGCGCTATCCT	C-terminal GFP fusion under native promoter
trpC(t)_rev	TGGGGGGAGTTTAGGGAAA	Backbone amplification
p5407_1.7kb_trpCT_OL_fwd	ATGCTCTTTCCCTAAACTCCCCCCAGGCACTTTTGTGTAATTGCGC	C-terminal mCherry fusion under native promoter

5407_mCherry_OL_rev	TTACTTACCTCGCCCTTGCTTACcttaattaaGGCTTCCTCGTCG	C-terminal mCherry fusion under native promoter
mCherry_5407_OL_fwd	CCCGACGAGGAAGCCttaattaagGTAAGCAAGGGCGAGGTAA	C-terminal mCherry fusion under native promoter
mCherry_tgluC_OL_rev	AATCATACATCTTATCTACATACGCTAAGCGGCCGCTTTGTAG	C-terminal mCherry fusion under native promoter
tgluC_for	CGTATGTAGATAAGATGTATGATT	Backbone amplification
pcol-19_pSW49_fwd	CGGGCCTCTTCGCTATTACGCCAGTacgtaccattattcgagacaac	Col-19 promoter
pcol-19_pSW49_rev	TCCGACGTCCCCAGGCAGAATGGCGgttgatgaactgatgtctttctaa	Col-19 promoter
ppf37_bb_rev	gagcaaagtgtttcccaactg	Backbone amplification
ppf37_bb_fwd	TCA GGT GGA TCT GGA GGC T	Backbone amplification
scar_col-19P_OL_fwd	gaaagacatcagttcatcaacATGGTCAGCAAGGGAGAGG	*MScarlet under col-19 in C. elegans*
scar_Pst_unc54_3UTR_rev	gcggccgatgcggagctcCTGCAGttaCTTGTAGAGCTCGTCCATTC	*MScarlet* in *C. elegans*
nipA_Stop_unc-54_OL_rev	tgacagcggccgatgcggagctcCTAGGCTTCCTCGTCGGG	NipA in *C. elegans*
nipA_unc-54_3'UTR_OL_rev	ccgatgcggagctcGAATTCGGCTTCCTCGTCGGGGAT	NipA in *C. elegans*
nipA_SP_col-19_OL_fwd	ttagaaagacatcagttcatcaacATGAAAGGCGCTATCCTCATTAT	*NipA in C. elegans under col-19*
col-19(P)_rev	gttgatgaactgatgtctttctaaatg	Backbone amplification
nipA_noStopp_rev	GGCTTCCTCGTCGGGGAT	*NipA in C. elegans*
nipA_SP_fwd	ATGAAAGGCGCTATCCTCATTA	NipA amplification
OL_col19p_nipA-ohneSP_fwd	gaaagacatcagttcatcaacATGCCGACCTCCAAAATATGC	*NipA in C. elegans under col-19*
peft-3_pPF37_OL_fwd	ggataacaatttcacaGGGCCCgcacctttggtcttttattgtcaac	*NipA in C. elegans under eft-3*
peft-3_nipA_OL_rev	GATAATGAGGATAGCGCCTTTCATgagcaaagtgtttcccaactg	*NipA in C. elegans under eft-3*
peft-3_*mScarlet*_OL_rev	ACTGCCTCTCCCTTGCTGACCATgagcaaagtgtttcccaactg	*NipA in C. elegans under eft-3*

dpy-7P_ppf37_OL_fwd	cggataacaatttcacaGGGCCCtcgaaagtct ctccggtag	*NipA in C. elegans under dpy-7*
dpy-7P_nipA_OL_rev	CTCCCTTGCTGACCATACCGGTTTCTCG ATGAAATCTCAACTTATATG	*NipA in C. elegans under dpy-7*
dpy-7P_scar_OL_rev	GTTGACCTCCACTAGCATTACACTTCTT GAAAGAGTGTCTCGGTTTT	*mScarlet expression under dpy-7*
